# Decreased Chloride Channel Expression in the Dorsolateral Prefrontal Cortex in Schizophrenia

**DOI:** 10.1371/journal.pone.0123158

**Published:** 2015-03-31

**Authors:** Courtney R. Sullivan, Adam J. Funk, Dan Shan, Vahram Haroutunian, Robert E. McCullumsmith

**Affiliations:** 1 University of Cincinnati College of Medicine, Neuroscience Graduate Program, Cincinnati, Ohio, United States of America; 2 Department of Psychiatry and Behavioral Neuroscience, University of Cincinnati, Cincinnati, Ohio, United States of America; 3 Department of Nephrology, University of Alabama Birmingham, Birmingham, Alabama, United States of America; 4 Department of Psychiatry, Mount Sinai School of Medicine, New York, New York, United States of America; 5 James J Peters Veterans Affairs Medical Center, New York, New York, United States of America; UTHSCSH, UNITED STATES

## Abstract

Alterations in GABAergic neurotransmission are implicated in several psychiatric illnesses, including schizophrenia. The Na-K-Cl and K-Cl cotransporters regulate intracellular chloride levels. Abnormalities in cotransporter expression levels could shift the chloride electrochemical gradient and impair GABAergic transmission. In this study, we performed Western blot analysis to investigate whether the Na-K-Cl and K-Cl cotransporter protein is abnormally expressed in the dorsal lateral prefrontal cortex and the anterior cingulate cortex in patients with schizophrenia versus a control group. We found decreased K-Cl cotransporter protein expression in the dorsal lateral prefrontal cortex, but not the anterior cingulate cortex, in subjects with schizophrenia, supporting the hypothesis of region level abnormal GABAergic function in the pathophysiology of schizophrenia. Subjects with schizophrenia off antipsychotic medication at the time of death had decreased K-Cl cotransporter protein expression compared to both normal controls and subjects with schizophrenia on antipsychotics. Our results provide evidence for KCC2 protein abnormalities in schizophrenia and suggest that antipsychotic medications might reverse deficits of this protein in the illness.

## Introduction

Schizophrenia is a complex and disabling illness characterized by impairments in attention, cognition, planning, and social function [1]. The schizophrenia phenotype may include positive, negative, and cognitive symptoms. Examples of these symptoms may include auditory hallucinations, social withdrawal, and working memory deficits, respectively [2–6]. Many of the cognitive and negative symptoms emerge from dysregulation of the prefrontal cortex (PFC) [7–9]. Recent evidence suggests these deficits may be secondary to alterations in the regulation of dorsolateral prefrontal cortex (DLPFC) pyramidal neurons by gamma-aminobutyric acid (GABA) interneurons.

Injection of GABA antagonists into DLPFC yields deficits in working memory similar to those found in schizophrenia [10, 11]. In individuals with schizophrenia, there is diminished parvalbumin (PV) expression in the DLPFC, a marker present in around 25% of GABAergic neurons [10, 12, 13]. However, the overall density of PV positive neurons is not changed, suggesting a functional impairment in GABAergic neurons in schizophrenia [10]. Moreover, studies have shown increased (>100%) GABAA alpha2 subunit expression on the axon initial segment of pyramidal neurons, without an increase in the pyramidal neurons themselves [10, 14]. This up-regulation could be a compensatory mechanism due to reduced inhibitory input from presynaptic GABAergic terminals [10]. Taken together, these data indicate a disturbance of inhibitory GABAergic neurons in this illness [15].

GABAergic interneuron function is mediated, in part, by chloride channels. The efficiency of GABAergic neurotransmission relies on the balance of intracellular chloride concentrations in the postsynaptic cell. Two chloride cotransporters, Na-K-Cl cotransporter (NKCC1) and K-Cl cotransporter (KCC2), are responsible for uptake and release of chloride ions, respectively [16–18]. Thus, we investigated chloride channel protein expression levels to assess the roles of these molecules in severe mental illness. Specifically, we hypothesize that abnormalities in the expression of these proteins may contribute to the pathophysiology of schizophrenia.

## Methods

### Subjects and Tissue Preparation

Anterior cingulate cortex (ACC) and DLPFC postmortem brain samples were provided by the Mount Sinai Medical Center and Bronx Veterans Administration Medical Center Brain Bank and consisted of thirty-four subjects with schizophrenia and twenty-nine nonpsychiatrically ill comparison subjects. Subjects were diagnosed with schizophrenia based on Diagnostic and Statistical Manual of Mental Disorders III Revision (DSM-III-R) criteria. The medical records of the subjects were examined using a formal blinded medical chart review instrument, as well as in person interviews with the subjects and/or their caregivers. The subjects were evaluated for National Institute of Neurological Disorders and Stroke and Association Internationale pour la Recherché et l'Enseignement en Neurosciences (NINDS-AIREN) criteria for a diagnosis of vascular dementia; National Institute of Neurological and Communicative Disorders and Stroke (NINCDS), DSM-IV and Consortium to Establish a Registry for Alzheimer's Disease (CERAD) diagnosis of dementia; Consensus criteria for a clinical diagnosis of Probable or Possible diffuse Lewy body disease; unified Parkinson's disease rating scale (UPDRS) for Parkinson’s disease; clinical criteria for diagnosis of Frontotemporal dementia; medical history of psychiatric disease; history of drug or alcohol abuse; and other tests of cognitive function including the mini–mental state examination (MMSE) and clinical dementia rating (CDR). In addition, each brain tissue specimen was examined neuropathologically using systematized macro- and microscopic evaluation using CERAD guidelines. Since the patients in our cohort were elderly at the time of death, many of the subjects have the cognitive impairment associated with aged subjects with schizophrenia [19–22]. All samples were derived from the left side of the brain. Subjects with schizophrenia were diagnosed with this illness for at least 30 years.

Subjects were excluded for a history of alcoholism, death by suicide, or coma for more than 6 hours before death. Next of kin consent was obtained for each subject [23]. Schizophrenia and comparison groups were matched for sex, age, pH, and PMI (Table 1). Information regarding the rationale for starting or stopping medications prior to death was not available. The 6 week cut off prior to death for on or off antipsychotic medications is based on reports of the effects of haloperidol on the brain lasting about 4–6 weeks in rodents after antipsychotics are stopped [24, 25]. The range of the time off antipsychotic medication for at least 6 weeks was 6–246 weeks, with the mean of 73 weeks, 6 days. We were unable to assess the effects of other psychotropic medications, such as benzodiazepines (BZD) or opiates, as we did not have enough subjects on these compounds to perform valid statistical tests (Table 1). Patients did not have access to alcohol or drugs of abuse. They did have access to tobacco, but data regarding tobacco use are not available for this cohort.

**Table 1:** Subjects’ Characteristics.

	DLPFC NKCC1		DLPFC KCC2		ACC KCC2	
Category	Comparison	Schizophrenia	Comparison	Schizophrenia	Comparison	Schizophrenia
n	28	34	28	32	29	33
Sex	11 m / 17 f	23 m / 11 f	11 m / 17 f	21 m / 11 f	12 m / 17 f	22 m / 11 f
AOD (years)	78 ± 15	74 ± 12	79 ± 14	73 ± 11	78 ± 14	74 ± 11
Tissue pH	6.4 ±. 2	6.4 ±. 3	6.4 ±. 3	6.3 ±. 3	6.4 ±. 2	6.3 ±. 3
PMI (hours)	7.8 ± 6.9	12.3 ± 6.5	8.3 ± 7.1	11.9 ± 6.6	7.5 ± 6.5	12.4 ± 6.9
APD on/off/unknown	0/28/0	23/11/0	0/28/0	23/9/0	0/29/0	23/10/0
BZD on/off/unknown	0/28/0	6/28/0	0/28/0	6/26/0	1/28/0	7/26/0
OP on/off/unknown	8/20/0	0/34/0	8/20/0	0/32/0	8/21/0	0/33/0

Abbreviations: female (f); male (m); age of death (AOD); post mortem interval (PMI); antipsychotic drugs (APD), benzodiazepine (BZD), opiates (OP), “off” refers the subject being off the medication for at least 6 weeks prior to death., “on” refers to being on the medication within 6 weeks of death. Data expressed mean +/- standard deviation.

For western blot studies, the ACC was dissected from Brodmann areas 24 and 32. The DLPFC was dissected from Brodmann areas 9 and 46. Samples were then stored at −80°C. Frozen tissue was first pulverized then homogenized in 300 μl of homogenization buffer containing 5mM Tris-HCl pH 7.4, 320 mM sucrose, and protease inhibitor tablet (Roche Diagnostics, Mannheim, Germany). All subjects were analyzed individually (they were not pooled).

### Antibodies

Commercial primary antibodies for human subjects were used as described below: NKCC1 (#P55011, UniProt, 1:500; Millipore, Billerica, Massachusetts, USA) and KCC2 (#Q9H2X9, UniProt, 1:1,000; Millipore, Billerica, Massachusetts, USA). Valosin-containing protein (VCP) (1:10,000; Novus Biological, Littleton, Colorado, USA) was used as a loading control.

Commercial primary antibodies for rodent studies were used as described below: KCC2 (1:2,000; Millipore, Billerica, Massachusetts, USA) and KCC2 (1:2000; Abcam, Cambridge, Massachusetts, USA). Valosin-containing protein (VCP) (1:10,000; Novus Biological, Littleton, Colorado, USA) was used as a loading control.

**Fig 1:**
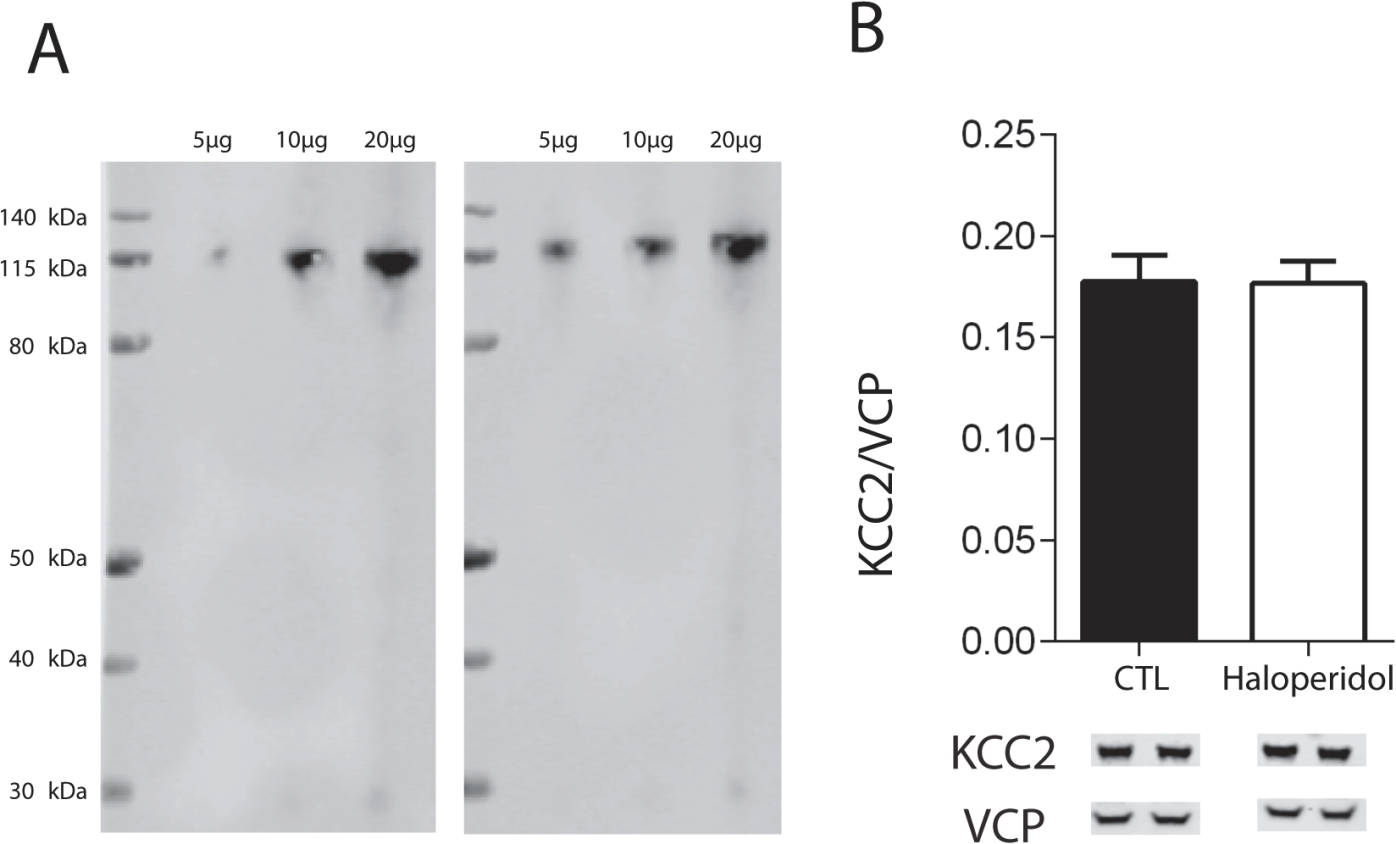
KCC2 Antibody Specificity in Rat. Expression of K-Cl cotransporter (KCC2) in rat prefrontal cortex with varying protein concentration (5 μg, 10 μg, 20 μg). Two different antibodies gave similar bands for KCC2 at the predefined molecular weight (123–126 kDa) (A). Expression of K-Cl cotransporter (KCC2) in prefrontal cortex in rats treated with haloperidol or vehicle for 9 months (B). Data are shown as mean ± SEM.

Two different antibodies gave similar bands for KCC2 at the predicted molecular weight (123–126 kDa) and no other bands were on the gel (Fig 1A).

### Human Western Blot Analysis

Postmortem brain tissue was homogenized with a large handheld homogenizer in 300 μl of homogenization buffer and aliquoted mixture into 50 μl tubes. Total protein concentration was determined with a bicinchoninic acid protein assay kit (Pierce Biotechnology, Inc., Rockford, Illinois, USA), and absorbance was measured on a SpectraCount absorbance microplate reader (Packard/Perkin Elmer, Wellesley, Massachusetts, USA) at 562 nm. Homogenates were stored in 50 μl aliquots at −80°C until assayed.

Samples for Western blot analyses were prepared with milli-Q water and sample buffer (6 X solution: 4.5% sodium dodecyl sulfate (SDS), 15% b-mercaptoethanol, 0.018% bromophenol blue, and 36% glycerol in 170mM Tris-HCl, pH 6.8) and heated at 70°C for 10 min. Samples (10 μl/well) were then run on 17 well 4–12% gradient gels. The same amount of protein (20 μg) was loaded for each sample. 14 wells were loaded per gel, balanced for control and schizophrenia in duplicate within gels. Investigators were not blinded to group identity. Gels were then transferred to PVDF membranes by BioRad semi-dry transblotters (Bio-Rad, Hercules, CA, USA). The membranes were blocked at 4°C in cold room overnight with LiCor Odyssey blocking buffer (LiCor, Lincoln, NE, USA) for all antibodies. After three 10 min washes in 1 X PBS with 0.1% Tween, the membranes were then incubated with the appropriate second antibody with IR-Dye 680 or 800cw labeled in LiCor blocking buffer with 0.2% Tween and SDS for 1 h at room temperature. Washes were repeated after secondary antibody incubation with 1 X PBS block. Membranes were scanned using a LiCor Odyssey scanner, and the intensity value for each protein band was measured using the Odyssey 3.0 software. We tested each antibody using varying concentrations of total protein from homogenized human cortical tissue to confirm we were in the linear range of the assay.

We initially measured NKCC1 and KCC2 in the DLPFC. We found a significant change in KCC2 in this region, and then measured KCC2 in the ACC to determine if this change was region specific.

### Rat Western Blot Analysis

Rodent studies were performed in accordance with the IACUC guidelines at the University of Alabama at Birmingham. Adult male Sprague-Dawley rats (250 g) were housed in pairs and maintained on a 12 hour light/dark cycle. Rats received 28.5 mg/kg haloperidol-decanoate or vehicle (sesame oil) by intramuscular injection every 3 weeks for 9 months. This dose was chosen based on previous reports [25–29]. Brain tissue was dissected and stored at -80°C.

Rat brain tissue sections were scraped from slides using a razor blade and homogenization buffer. Total protein concentration was determined with a bicinchoninic acid protein assay kit (Pierce Biotechnology, Inc., Rockford, Illinois, USA), and absorbance was measured on a SpectraCount absorbance microplate reader (Packard/Perkin Elmer, Wellesley, Massachusetts, USA) at 562 nm. Homogenates were stored in 50 μl aliquots at −80°C until assayed.

Samples for Western blot analyses were prepared with milli-Q water and sample buffer (6 X solution: 4.5% sodium dodecyl sulfate (SDS), 15% b-mercaptoethanol, 0.018% bromophenol blue, and 36% glycerol in 170mM Tris-HCl, pH 6.8) and heated at 70°C for 10 min. Samples (10 μl/well) were then run on 4–12% gradient gels. The same amount of protein (10 μg) was loaded for each sample. Seventeen samples were loaded per gel, alternating treated and untreated in duplicate within gels. Investigators were not blinded to group identity. Gels were then transferred to PVDF membranes by BioRad semi-dry transblotters (Bio-Rad, Hercules, CA, USA). The membranes were blocked for 1 hour at room temperature with LiCor Odyssey blocking buffer (LiCor, Lincoln, NE, USA) and incubated at 4°C in cold room overnight with for all antibodies. After three 10 min washes in 1 X PBS with 0.1% Tween, the membranes were then incubated with the appropriate second antibody with IR-Dye 680 or 800cw labeled in LiCor blocking buffer with 0.2% Tween and SDS for 1 hour at room temperature. Washes were repeated after secondary antibody incubation with 1 X PBS block. Membranes were scanned using a LiCor Odyssey scanner, and the intensity value for each protein band was measured using the Odyssey 3.0 software. We tested each antibody using varying concentrations of total protein from homogenized rat cortical tissue to confirm we were in the linear range of the assay.

### Data Analysis

Using Odyssey Version 3.0 analytical software, near-infrared emissions detected by the LiCor Odyssey scanner were expressed as integrated intensity with top-bottom median intra-lane background subtraction. In each subject, duplicate lanes of NKCC1 and KCC2 protein expression were normalized to VCP as an in-lane loading control and then averaged for each subject. VCP was chosen as a loading control because VCP was previously determined to be unchanged in schizophrenia compared to control subjects [30].

Data were analyzed using Statistica (Statsoft, Tulsa, Oklahoma, USA). Correlation analyses were performed to probe for associations between the expression of NKCC1 or KCC2 and tissue pH, age, and postmortem interval. One-way analysis of variance was used for all human analyses. Analysis of variance was performed to assess the effects of antipsychotic medication exposure in subjects with schizophrenia and in rats treated with antipsychotic medications.

## Results

**Fig 2:**
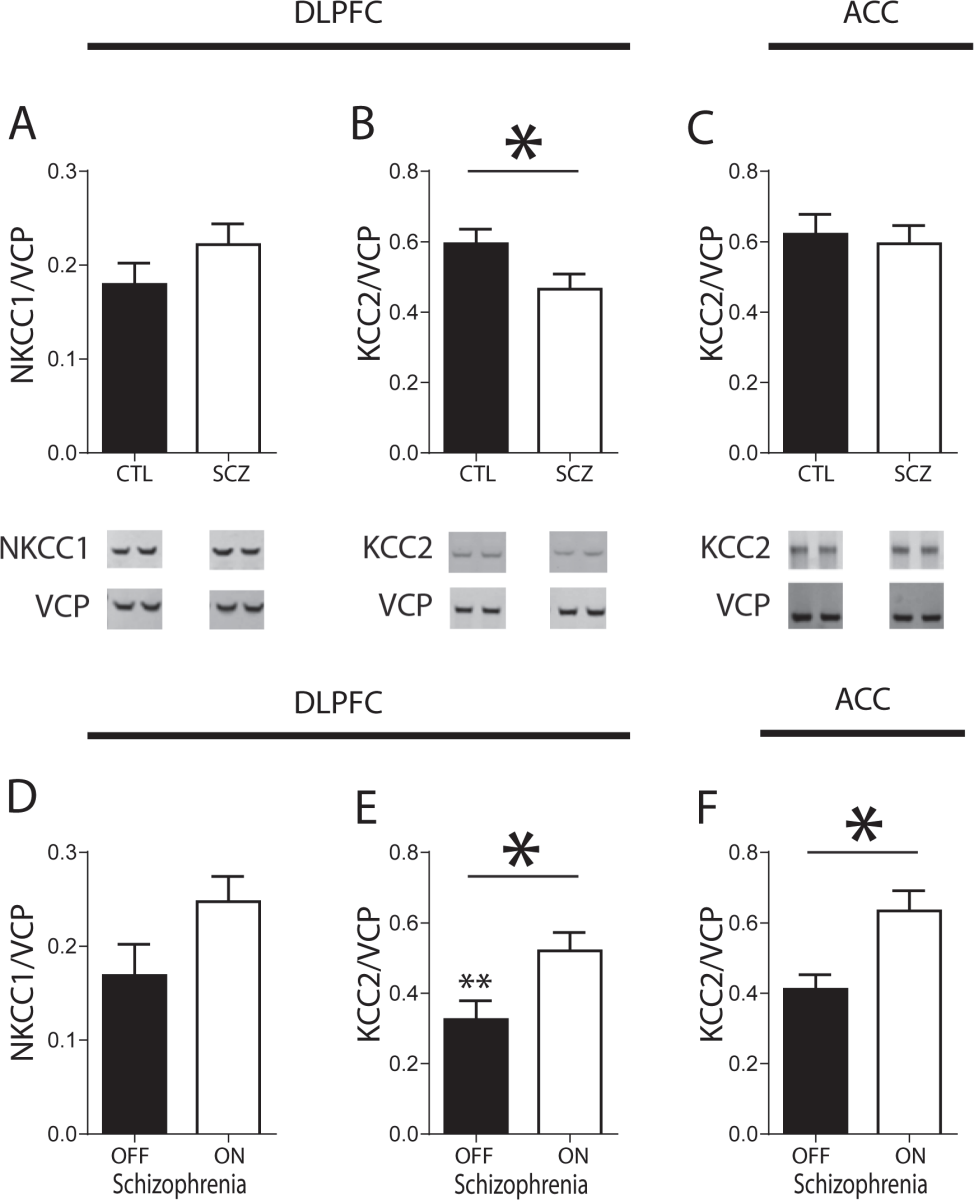
NKCC1 and KCC2 Expression in DLPFC. Expression of Na-K-Cl cotransporter (NKCC1) (A, D) and K-Cl cotransporter (KCC2) (B, E) protein in the DLPFC in control subjects (CTL) and subjects with schizophrenia (SCZ) (A, B) and in subjects with schizophrenia on and off medication for at least 6 weeks (D, E). Expression of KCC2 protein in the ACC in control subjects (CTL) and subjects with schizophrenia (SCZ) (C) and in subjects with schizophrenia on and off medication for at least 6 weeks (F). *P<0.05 **P<0.05 compared to KCC2 expression in the DLPFC of control subjects. Data are shown as mean ± SEM.

No significant associations were found between pH, post-mortem interval (PMI) or age and any of our dependent measures. In subjects with schizophrenia, we found a 22% decrease in KCC2 protein expression in the DLPFC [F(1,58) = 2.140, p<0.05]. We did not detect changes in NKCC1 protein levels in this region (Fig 2A and 2B). To determine if changes in KCC2 expression were region-specific, we measured KCC2 protein levels in the ACC. We did not any detect changes in KCC2 expression in the ACC (Fig 2C).

In both the DLPFC and the ACC, subjects with schizophrenia off antipsychotics for 6 weeks or more at the time of death had a 38% and 35% decrease in KCC2 expression compared to subjects with schizophrenia on antipsychotics, respectively [F(1,30) = 2.159, p<0.05 and F(1,31) = 2.439, p<0.05] (Fig 2E and 2F). There was no significant change in NKCC1 between subjects with schizophrenia on and off medication in the DLPFC (Fig 2D). We also found a significant decrease in KCC2 expression in subjects with schizophrenia off medication compared to controls in the DLPFC [F(1,57) = 4.415, p<0.05].

We did not find any changes in KCC2 expression in rats treated with 28.5 mg/kg haloperidol-decanoate or vehicle every 3 weeks for 9 months [F(1,18) = 0.05611, p = 0.9559] (Fig 1B).

## Discussion

While multiple markers of GABAergic neurotransmission are abnormal in schizophrenia, the contribution of chloride channels to these irregularities is less well understood [10, 31–33]. Alterations in expression levels of chloride transporter regulatory kinases could shift the chloride electrochemical gradient and ultimately impair GABA transmission in schizophrenia [32]. Previous studies found that messenger RNA (mRNA) expression levels of "Oxidative Stress Response kinase" (OXSR1) and "With No lysine Kinase 3" (WNK3) were increased in schizophrenia in the DLPFC [32]. Maintenance of the electrochemical gradient is dependent on the phosphorylation or dephosphorylation of cotransporter proteins by these regulatory kinases [18]. Changes in NKCC1 or KCC2 activity alone may result in unbalanced intracellular chloride levels, leading to abnormal GABAergic function [32, 34]. NKCC1 is phosphorylated and activated by OXSR1, resulting in an increase in intracellular chloride [32]. Another regulatory kinase, WNK3, both activates NKCC1 and inhibits KCC2, also resulting in increased intracellular chloride [35]. The authors of this study proposed that increases in these kinases could disrupt the balance of chloride transport by increasing intracellular chloride levels in GABAergic targets [10]. No change in cotransporter mRNA was found in this study, suggesting that changes in expression of these regulatory kinases do not directly impact cotransporter gene expression [32]. However, lower KCC2 activity could diminish the extrusion of chloride from GABAergic target neurons, resulting in GABA channels on the postsynaptic membrane allowing passive outflow of Cl- and an excitatory effect upon GABA binding [32, 35, 36]. This could create diminished GABAergic inhibitory tone in subjects with schizophrenia following GABA binding [36].

In the present study, we found decreased levels of KCC2 protein expression in the DLPFC in schizophrenia. Interestingly, KCC2 is exclusively expressed in neurons, while NKCC1 is localized to glia [37–40]. Lower KCC2 protein levels would further reduce the extrusion of chloride from GABAergic neurons, leading to an imbalance of intracellular chloride levels that could also lead to poor efficiency of GABA channels [32, 41]. Taken together, these findings are consistent with a pathophysiologic process suggesting increased intracellular chloride levels in schizophrenia. These data support the hypothesis that a disruption of intraneuronal chloride homeostasis may contribute to altered GABAergic function in the DLPFC in schizophrenia.

We found decreased KCC2 protein expression in the DLPFC but not in the ACC. This suggests a region specific down-regulation of KCC2 in schizophrenia. Numerous studies have found region-specific gene expression in schizophrenia, possibly due to differences in neurocircuitry between the ACC and DLPFC [42–45]. Studies have shown a loss of interneurons in the ACC in subjects with schizophrenia [46, 47] as well as a loss of serotonin 5HT2 receptor binding sites on GABAergic interneurons. These changes may lead to altered GABAergic inhibitory regulation of ACC pyramidal cells, and possibly contribute to the pathophysiology of schizophrenia [48]. We hypothesize our findings contribute to the GABA-related changes in the prefrontal cortex, specifically in the DLPFC.

We also found that subjects with schizophrenia who were off antipsychotic medications for 6 weeks or more at the time of death had decreased KCC2 expression in the ACC and DLPFC, compared to those who were on antipsychotic medications. This decrease in schizophrenia subjects off medication suggests that expression of chloride channels might be modulated via dopaminergic systems and D2 antagonists. To further assess the effects of antipsychotic medication, we treated rats for 9 months with haloperidol-decanoate (28.5 mg/kg every 3 weeks) or vehicle (sesame oil) to simulate a lifetime of antipsychotic treatment. We chose the antipsychotic haloperidol because nearly all of our subjects with schizophrenia were taking typical antipsychotics. We did not find any changes in KCC2 expression in the haloperidol treated rats, suggesting that the decrease in KCC2 protein expression is not due to a medication effect, and that the absence of a decrease in KCC2 protein levels in medicated subjects with schizophrenia is a disease by treatment interaction (Fig 1B).

While very few subjects were taking BZDs or opioids at the time of death, it is worthwhile noting the potential effects these drugs might have on related neural networks. Anxiety associated with schizophrenia may be attenuated with the use of benzodiazepines; however, their efficacy as an anxiolytic varies widely and they are used on a limited basis [49, 50]. BZDs work by increasing the inhibitory effects GABAergic neurons have on their targets [51, 52]. After GABA receptor binding, they modulate the GABAA receptor complex allosterically, increasing total chloride ion flux across the postsynaptic membrane. This could lead to stronger GABAergic tone and hyperpolarization of the GABAergic target, reversing the possible effects of the changes in chloride transporter expression we observed in subjects with schizophrenia. In contrast, opioid use could potentially antagonize the inhibitory effects of GABA [53]. Studies have shown that opioid binding to the mu-opioid receptor results in a decrease in presynaptic vesicular GABA release and inhibition of GABAergic neurotransmission [53–55]. Subjects taking opioids at the time of death could have an exacerbation of diminished inhibitory tone exerted by GABA. Finally, we were unable to account for the putative effects of nicotine as smoking data were not available for the study cohort.

Our present findings of decreased KCC2 protein in the DLPFC varies from one previous report that did not find changes in KCC2 mRNA [32]. These divergent findings could be secondary to factors such as age, PMI, and/or antipsychotic medication treatment that vary between the two studies, which utilized tissues from different brain collections. For example, our subjects were on average 20 years older, with PMIs about half of the previous work [32]. In addition, while the prior study measured mRNA, we measured protein levels, which do not always have the same valence of change as one another [45, 56]. Postmortem studies commonly present challenges such as these, necessitating development of more innovative techniques, and larger, better characterized cohorts of subjects [57, 58].

Taken together with previous findings of altered GABA signaling in this illness, our data support the hypothesis that decreased KCC2 expression in the DLPFC contributes to the pathophysiology of schizophrenia. Our data also support the conclusion that decreases in KCC2 protein expression are not a medication effect, and raise the possibility that antipsychotic medications might restore expression of this protein in subjects with schizophrenia.
